# Clinical outcomes according to cannula configurations in patients with acute respiratory distress syndrome under veno-venous extracorporeal membrane oxygenation: a Korean multicenter study

**DOI:** 10.1186/s13613-020-00700-9

**Published:** 2020-06-22

**Authors:** Sung Yoon Lim, Soyeon Ahn, Sang-Bum Hong, Chi Ryang Chung, Kyeongman Jeon, Sang-Min Lee, Woo Hyun Cho, Sunghoon Park, Young-Jae Cho

**Affiliations:** 1 Division of Pulmonary and Critical Care Medicine, Department of Internal Medicine, Seoul National University College of Medicine, Seoul National University Bundang Hospital, Seongnam-Si, Gyeonggi-Do Republic of Korea; 2 Medical Research Collaborating Center, Seoul National University Bundang Hospital, Seongnam-Si, Gyeonggi-Do Republic of Korea; 3 Department of Pulmonary and Critical Care Medicine, Asan Medical Center, Seoul, Republic of Korea; 4 Department of Critical Care Medicine, Samsung Medical Center, Seoul, Republic of Korea; 5 Division of Pulmonary and Critical Care Medicine, Department of Internal Medicine, Seoul National University Hospital, Seoul, Republic of Korea; 6 Department of Internal Medicine, Pusan National University Yangsan Hospital, Yangsan-Si, Gyeongsangnam-do Republic of Korea; 7 Division of Pulmonary and Critical Care Medicine, Department of Medicine, Hallym University Sacred Heart Hospital, Anyang-Si, Gyeonggi-do Republic of Korea

**Keywords:** Acute respiratory distress syndrome, PaO_2_, Recirculation, Veno-venous extracorporeal membrane oxygenation, Complications

## Abstract

**Background:**

Recirculation during veno-venous extracorporeal membrane oxygenation (VV-ECMO) is a known drawback that limits sufficient oxygenation. This study aimed to compare the short-term oxygenation and long-term mortality based on cannula configuration in patients with acute respiratory distress syndrome (ARDS) who receive VV-ECMO, especially in the absence of newly developed dual-lumen, single cannula.

**Methods:**

Data of patients with severe ARDS who received VV-ECMO from 2012 to 2015 at six hospitals were retrospectively analyzed. Primary outcomes were the partial pressure of oxygen (PaO_2_) at 1, 4, and 12 h after ECMO initiation and 180-day mortality.

**Results:**

Patients (*n* = 335) were divided into two groups based on the return cannula site: femoral vein (*n* = 178) or internal jugular vein (*n* = 157). The propensity score matching analysis generated 90 pairs, and baseline characteristics at admission, including PaO_2_, were similar between the groups. PaO_2_ at 1, 4 and 12 h after ECMO initiation were not different according to cannula configuration. Moreover, the increment in oxygenation from the baseline values was not different between the femoral and jugular group. PaCO_2_ level at 1, 4 and 12 h were significantly lower in the jugular group. The two groups did not differ in terms of mortality at 180 days after ECMO, however more cannula-related complications occurred in the jugular group.

**Conclusion:**

Regardless of the cannula configuration, patients with ARDS managed with VV-ECMO showed comparable clinical outcomes in terms of short-term oxygenation and long-term mortality. Nevertheless, further well-designed randomized control trials are warranted.

## Background

Veno-venous extracorporeal membrane oxygenation (VV-ECMO) is used as a rescue therapy in patients with acute respiratory failure when mechanical ventilation is not sufficient to maintain adequate oxygenation or CO_2_ elimination [1, 2]. Recently, single cannula has been used for VV-ECMO support, however classic configurations composed by cannulation of two vessels (double cannulation) is still used in many countries: one for draining the blood from the venous system to the ECMO circuit, and the other for returning the oxygenated blood to the right atrium [3]. Two types of classic configuration are identified by the cannulation site of the return catheter. Both configurations commonly insert drainage catheters through the femoral vein, but the return catheter can be either through the jugular vein (fem–jug) or the other femoral vein (fem–fem configuration).

Both femoral veins are usually large and easily accessible for rapid access and initiation of VV-ECMO with fem–fem configuration [4]. However, the return blood in this configuration is directed toward the superior vena cava (SVC) rather than the tricuspid valve, potentially creating abnormal flows away from the valve and possibly increasing recirculation [3, 5, 6]. The fem–jug configuration potentially has less recirculation and may enable higher blood flows than fem–fem configuration, because the blood is directed toward the tricuspid valve. Thus, the fem–jug configuration is increasingly preferred lately [7]. The recently published EOLIA trial on VV-ECMO shows similar trends, in which cannulation was performed with a fem–jug configuration in 95% of the patient population [8].

However, the validity of this assumption is yet to be evaluated, and its implications on the patients’ outcomes need to be assessed. The primary objective of this study was to compare the short-term oxygenation and long-term mortality according to cannula configuration in patients with acute respiratory distress syndrome (ARDS) treated with VV-ECMO, especially in the absence of the newly developed dual-lumen, single cannula.

## Methods

### Study design

This multicenter study included patients with acute respiratory failure who did not respond to conventional treatment and hence received ECMO therapy in South Korea. The data of all patients who required VV-ECMO support at the six major ECMO centers from January 2012 to December 2015 were included and retrospectively analyzed. Patients who met the following criteria were excluded: (1) age under 18 years, (2) ECMO support for less than 24 h, (3) death within 2 days from ECMO initiation, (4) use of VA ECMO or three cannulas: VVA and VAV, (5) underwent bridge to lung transplantation, and (6) incomplete data. The study was approved by the Institutional Review Board of The Seoul National University Bundang Hospital and by the local institutional review boards of all other participating centers. The requirement for informed consent was waived considering the retrospective nature of the study.

### Data collection

After a review of the electronic medical records, clinical data were collected using a standardized registry form. The registry form included demographic information, Acute Physiology, and Chronic Health Evaluation (APACHE) II and Sequential Organ Failure Assessment (SOFA) scores calculated using the worst value within 24 h of intensive care unit (ICU) admission, and the etiology of respiratory failure. Information on adjunctive therapy such as the use of vasopressors, steroids, neuromuscular blockade, prone positioning, nitric oxide, bicarbonate infusion, and continuous renal replacement therapy (CRRT) were collected. Data on the pre-ECMO hemodynamic parameters, pre- and post-ECMO ventilator settings, and arterial blood gas prior to ECMO initiation were collected. The ECMO parameters included in the registry were duration of ECMO, duration from mechanical ventilation to ECMO initiation, hospital stay, and weaning success from ECMO. Weaning success from ECMO was defined as survival after 48 h of ECMO decannulation. Distance between the tips of drainage and infusion cannula was measured using chest radiography performed at the end of the procedure.

### Clinical outcomes

The main outcome of our study was the arterial partial pressure of oxygen (PaO_2_) immediately, at 4 h, and 24 h after ECMO initiation. Other outcome variables were cannula-related complications and 90-day or 180-day mortality. Cannula-related complications included: (1) bloodstream infection (BSI) during ECMO support, defined as a case with confirmed organisms from one or more blood cultures during the period 48 h after the initiation of ECMO to 24 h after ECMO weaning; (2) ECMO catheter-related BSI was defined as a confirmed BSI without a definite source of infection except the ECMO catheter [9]; (3) cannula-related bleeding events were defined according to the Extracorporeal Life Support Organization (ELSO) definition [10], as clinically overt bleeding from the cannula site recorded in the medical and/or nursing charts associated with either administration of two or more RBC units in 24 h or a drop in hemoglobin greater than 2 g/L over 24 h, or if bleeding required an intervention [11].

### Statistical analysis

Normally distributed variables are presented as mean (SD) and compared using an independent or paired *t* test as appropriate. Nonparametric continuous variables are presented as the median (interquartile range) and compared using an independent or paired Mann–Whitney U test as appropriate. Categorical variables are expressed as the number (percentage) and compared using Pearson’s Chi-square test or Fisher’s exact test. To assess the change in outcome measures over time, generalized estimating equations (GEEs) were used at every time point, with the baseline values as covariates. Survival curves and rates were obtained by Kaplan–Meier analysis and differences in survival rates were compared using the log-rank test. To reduce the effect of potential confounding effects between two groups, significant differences in baseline characteristics were adjusted by propensity score matching. We used nearest-neighbor matching scheme with a caliper size of 0.1 and matched the patients in a 1:1 ratio. We considered the covariate balance as achieved if the absolute standardized difference between the two groups was ≤ 0.2. All statistical analyses were performed using R, version 3.3.1, (R Foundation Inc; http://cran.r-project.org/). *P* values less than 0.05 were considered statistically significant.

## Results

### Baseline clinical characteristics of the study population

During the study period (2012–2015), ECMO support was provided to 445 patients in the participating six hospitals. After excluding 110 patients, we analyzed 335 (75.3%) patients who received VV-ECMO specifically for respiratory failure (Additional file 1). The patients were divided into two groups according to the site of infusion catheter: jugular (*n* = 157) or femoral (*n* = 178). Respiratory ECMO Survival Prediction (RESP) score was higher in the jugular compared to the femoral group (0.9 ± 3.2 vs. 0.6 ± 3.2, respectively; *P* < 0.001). Also, the patients were not equally distributed between the two groups with regard to each participating center (Table 1).

**Table 1:** Baseline characteristics according to the cannulation (before and after propensity score matching)

	Unmatched cohort				Matched cohort			
	Total (*n* = 335)	Jugular (*n* = 157)	Femoral (*n* = 178)	*P*	Total (*n* = 180)	Jugular (*n* = 90)	Femoral (*n* = 90)	*P*
Age	55.6 ± 14.7	55.5 ± 14.3	55.7 ± 15	0.885	56.7 ± 14	56.4 ± 14.2	57 ± 13.9	0.759
Sex	222 (66.3)	111 (70.7)	111 (62.4)	0.135	113 (62.8)	54 (60)	59 (65.6)	0.537
BMI	22.9 ± 4.1	23.1 ± 4.3	22.6 ± 3.9	0.281	22.6 ± 4.1	23 ± 4.4	22.2 ± 3.7	0.166
APACHE	22.1 ± 9.3	20 ± 8.8	24 ± 9.4	*< 0.001*	22.3 ± 9.4	21.8 ± 9.4	22.8 ± 9.4	0.507
SOFA	8.5 ± 4.3	8.6 ± 4.2	8.5 ± 4.5	0.812	8.6 ± 4.5	8.6 ± 4.4	8.7 ± 4.6	0.894
RESP score	0.2 ± 3.3	0.9 ± 3.2	0.6 ± 3.2	*< 0.001*	0 ± 3.1	0 ± 3	0 ± 3.1	0.846
Preserve score	5.4 ± 1.8	5.3 ± 1.9	5.5 ± 1.8	0.519	5.4 ± 1.8	5.3 ± 1.9	5.4 ± 1.7	0.726
Etiology of respiratory failure				*0.004*				0.192
Viral pneumonia	49 (14.6)	23 (14.6)	26 (14.6)		31 (17.2)	14 (15.6)	17 (18.9)	
Bacterial pneumonia	133 (39.7)	79 (50.3)	54 (30.3)		73 (40.6)	42 (46.7)	31 (34.4)	
COPD and asthma	4 (1.2)	3 (1.9)	1 (0.6)		1 (0.6)	0 (0.0)	1 (1.1)	
Trauma and burn	14 (4.2)	8 (5.1)	6 (3.4)		7 (3.9)	2 (2.2)	5 (5.6)	
Asphyxia	4 (1.2)	2 (1.3)	2 (1.1)		2 (1.1)	2 (2.2)	0 (0.0)	
Acute exacerbation of ILD	65 (19.4)	22 (14.0)	43 (24.2)		36 (20.0)	18 (20.0)	18 (20.0)	
Chronic respiratory failure	16 (4.8)	5 (3.2)	11 (6.2)		6 (3.3)	2 (2.2)	4 (4.4)	
Airway obstruction	25 (7.5)	6 (3.8)	19 (10.7)		15 (8.3)	4 (4.4)	11 (12.2)	
Other respiratory failure	25 (7.5)	9 (5.7)	16 (9.0)		9 (5.0)	6 (6.7)	3 (3.3)	
Pre-ECMO rescue therapy				1.000				0.765
NMB	179 (54.1)	83 (53.9)	96 (54.2)		95 (52.8)	46 (51.1)	49 (54.4)	
NO	103 (31.1)	25 (16.2)	78 (44.1)		51 (28.3)	25 (27.8)	26 (28.9)	
Prone	80 (25.1)	55 (38.7)	25 (14.1)		45 (25.6)	30 (34.9)	15 (16.7)	
Steroid	63 (19.6)	31 (21.7)	32 (18)		34 (19.3)	21 (24.4)	13 (14.4)	
CRRT	53 (15.8)	19 (12.1)	34 (19.1)		27 (15)	14 (15.6)	13 (14.4)	
MV	318 (94.9)	155 (98.7)	163 (91.6)		177 (98.3)	88 (97.8)	89 (98.9)	
Center				*<0.001*				*<0.001*
A	44 (13.1)	5 (3.2)	39 (21.9)		28 (15.6)	5 (5.6)	23 (25.6)	
B	109 (32.5)	86 (54.8)	23 (12.9)		68 (37.8)	50 (55.6)	18 (20)	
C	44 (13.1)	3 (1.9)	41 (23)		16 (8.9)	3 (3.3)	13 (14.4)	
D	77 (23)	18 (11.5)	59 (33.1)		33 (18.3)	10 (11.1)	23 (25.6)	
E	45 (13.4)	44 (28)	1 (0.6)		22 (12.2)	22 (24.4)	0 (0)	
F	16 (4.8)	1 (0.6)	15 (8.4)		13 (7.2)	0 (0)	13 (14.4)	
Year				0.216				0.633
2	76 (22.7)	34 (21.7)	42 (23.6)		45 (25)	23 (25.6)	22 (24.4)	
3	77 (23)	34 (21.7)	43 (24.2)		35 (19.4)	20 (22.2)	15 (16.7)	
4	87 (26)	36 (22.9)	51 (28.7)		51 (28.3)	22 (24.4)	29 (32.2)	
5	95 (28.4)	53 (33.8)	42 (23.6)		49 (27.2)	25 (27.8)	24 (26.7)	

Values are expressed as mean ± standard deviation, or *n* (%); significant *P* values are in italic

*APACHE* Acute Physiology and Chronic Health Evaluation, *SOFA* Sequential Organ Failure Assessment, *PRESERVE* Predicting Death for Severe Acute Respiratory Distress Syndrome on Veno-venous ECMO, *COPD* chronic obstructive pulmonary disease, *ILD* interstitial lung disease, *ARDS* acute respiratory distress syndrome, *ECMO* extracorporeal membrane oxygenation, *NMB* neuromuscular blockade, *NO* nitric oxide, *CRRT* continuous renal replacement therapy, *MV* mechanical ventilation, *BMI* body mass index

To reduce the effect of treatment-selection bias and potential confounding factors; we adjusted for age, sex, participating center and year; APACHE, SOFA, and Respiratory ECMO Survival Prediction (RESP Score); neuromuscular blockade, nitric oxide administration, use of CRRT, use of mechanical ventilation for pre-ECMO recue therapy, and immunocompromised status by propensity score matching analysis. The analysis generated 90 pairs, and the characteristics of the pairs were balanced with a standardized difference less than 20% for all baseline variables (Additional file 2). There were no significant differences in APACHE score, SOFA score, and RESP score between the two matched groups.

### Pre-ECMO parameters

Table 2 shows the baseline arterial blood gas analysis and the mechanical ventilator settings before the initiation of ECMO. No statistically significant differences were observed in PaO_2_, the fraction of inspired oxygen (FiO_2_), and its ratio, PaO_2_/FiO_2_, between the two groups. However, differences between groups still remained in the ventilatory parameters such as tidal volume and respiratory rate (jugular vs. femoral; 7.0 ± 2.9 vs. 8.1 ± 3.3; 22.7 ± 6.1 vs. 25.0 ± 7.0, respectively; *P* < 0.05), and partial pressure of carbon dioxide (PaCO_2_) were lower in the jugular group than in femoral group (51.3 ± 18.5 vs. 57.5 ± 18.7, respectively; *P* = 0.031).

**Table 2:** Pre-ECMO parameters of patients supported with ECMO for respiratory failure

	Unmatched cohort				Matched cohort			
	Total (*n* = 335)	Jugular (*n* = 157)	Femoral (*n* = 178)	*P*	Total (*n* = 180)	Jugular (*n* = 90)	Femoral (*n* = 90)	*P*
Ventilation parameters								
FiO_2_	0.9 ± 0.1	0.9 ± 0.1	0.9 ± 0.1	0.202	0.9 ± 0.1	0.9 ± 0.2	1.0 ± 0.1	*<0.001*
PIP (cmH_2_O)	28.6 ± 7.0	28.3 ± 7.5	28.9 ± 6.4	0.615	28.8 ± 6.5	28.1 ± 6.7	29.6 ± 6.1	*0.006*
PEEP (cmH_2_O)	9.0 ± 3.6	9.6 ± 3	8.3 ± 4.1	*0.030*	9.1 ± 3.6	9.2 ± 3.1	9.1 ± 4.1	0.683
TV (mL/kg)	7.5 ± 3.1	7.0 ± 2.9	8.1 ± 3.3	*0.036*	7.6 ± 3.2	7.0 ± 3.1	8.1 ± 3.3	*0.008*
Driving P (cmH_2_O)	19.6 ± 6.8	18.7 ± 7.1	20.6 ± 6.3	0.071	20.0 ± 6.7	19.2 ± 6.9	20.6 ± 6.5	*0.030*
MV (L/min)	10.3 ± 4.4	9.3 ± 3.9	11.6 ± 4.6	*0.001*	10.3 ± 4.4	9.0 ± 3.9	11.7 ± 4.5	*<0.001*
Respiratory rate	23.8 ± 6.7	22.7 ± 6.1	25 ± 7	*0.027*	24.0 ± 7.2	22.2 ± 6.4	25.7 ± 7.5	*<0.001*
Arterial blood gases								
pH	7.3 ± 0.2	7.3 ± 0.1	7.3 ± 0.2	0.090	7.3 ± 0.2	7.3 ± 0.1	7.3 ± 0.2	0.219
PaCO_2_ (mmHg)	54.4 ± 18.8	51.3 ± 18.5	57.5 ± 18.7	*0.031*	55.6 ± 23.7	54.9 ± 25.4	56.3 ± 22.0	0.620
PaO_2_ (mmHg)	68.0 ± 30.9	65.2 ± 23.1	70.8 ± 37.1	0.246	67.8 ± 31.9	67.0 ± 24.5	68.7 ± 37.6	0.641
HCO_3_ (mEq/L)	24.4 ± 7.4	24.3 ± 7.5	24.4 ± 7.4	0.895	25.0 ± 8.8	25.3 ± 8.4	24.7 ± 9.2	0.538
SaO_2_ (%)	85 ± 12.5	85.2 ± 12.1	84.7 ± 13	0.771	84.7 ± 13.0	85.3 ± 12.3	84.1 ± 13.7	0.410
PF ratio	75.6 ± 42.7	79.6 ± 45.2	71.7 ± 39.9	0.114	76.7 ± 48.2	76.5 ± 48.6	77.0 ± 48.1	0.948

Values are expressed as mean ± standard deviation; significant *P* values are in italic

*ECMO* extracorporeal membrane oxygenation, *FiO*_*2*_ fraction of inspired oxygen, *PIP* peak inspiratory pressure, *PEEP* positive end-expiratory pressure, *TV* tidal volume, *Driving P* driving pressure, *MV* minute ventilation, *PaCO*_*2*_ partial pressure of carbon dioxide, *PaO*_*2*_ partial pressure of oxygen, *HCO*_*3*_ bicarbonate, *SaO*_*2*_ oxygen saturation, *PF* PaO_2_/FiO_2_

### Oxygenation and ventilation profiles during ECMO

**Fig. 1:**
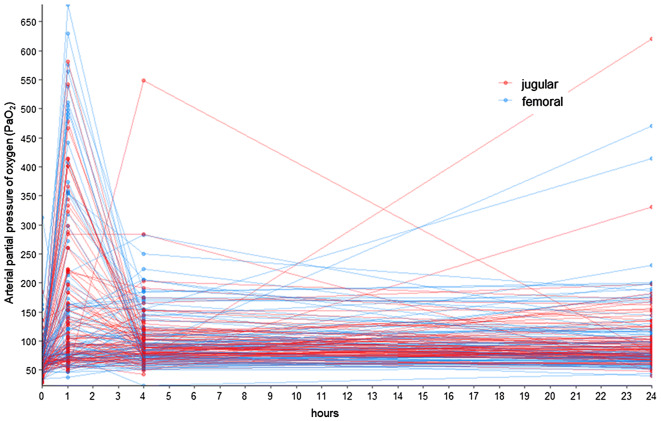
Change in the arterial partial pressure of oxygen (PaO_2_) during ECMO based on cannula configuration. Red line indicating jugular group in which return cannula site of VV-ECMO is femoral vein. Blue line depicting femoral group in which return cannula site of VV-ECMO is internal jugular vein

Figure 1 presents the PaO_2_ overtime after ECMO initiation among the matched cohort. In both femoral and jugular groups, the mean PaO_2_ appeared to increase after initiation of ECMO. The levels of arterial oxygenation at each time point after ECMO initiation were not different according to cannula configuration (Table 3, Additional file 3: Table S1). To analyze the effects of ECMO on oxygenation at each point, the GEE was used at all points, with the baseline values as covariates. GEE analysis revealed that oxygenation was significantly improved after ECMO initiation, however, change in oxygenation was not different between the femoral and jugular group. Whereas the PaCO_2_ level was significantly lower in the jugular group at baseline and at every time point after cannulation, the change in CO_2_ level was not different between groups as estimated by GEE analysis.

**Table 3:** Oxygenation and ventilation profiles during ECMO

	Total (*n* = 180)	Jugular (*n* = 90)	Femoral (*n* = 90)	*P*
Baseline				
PaCO_2_ (mmHg)	54.4 ± 18.8	51.3 ± 18.5	57.5 ± 18.7	*0.031*
PaO_2_ (mmHg)	68 ± 30.9	65.2 ± 23.1	70.8 ± 37.1	0.246
MV (L/min)	10.3 ± 4.4	9.3 ± 3.9	11.6 ± 4.6	*0.001*
PIP (cmH2O)	28.6 ± 7.0	28.3 ± 7.5	28.9 ± 6.4	0.615
Immediately after cannulation				
PaCO_2_ (mmHg)	34.7 ± 10.5	31.9 ± 9.3	37.5 ± 10.9	*<0.001*
PaO_2_ (mmHg)	170.4 ± 145.7	158.9 ± 125.2	182 ± 163.8	0.298
MV (L/min)	5.6 ± 4	4.5 ± 3	7.0 ± 4.6	*<0.001*
PIP (cmH2O)	22.8 ± 5.8	22.3 ± 5.1	23.2 ± 6.4	0.289
4 h after cannulation				
PaCO_2_ (mmHg)	33.4 ± 7.4	31.7 ± 6.8	35.2 ± 7.6	*0.002*
PaO_2_ (mmHg)	102.7 ± 55.9	102.4 ± 61.3	103.1 ± 50.2	0.930
MV (L/min)	4.2 ± 2.7	3.8 ± 2.3	4.7 ± 3.0	*0.034*
PIP (cmH2O)	21.2 ± 4.9	21.5 ± 4.8	20.9 ± 5.0	0.423
24 h after cannulation				
PaCO_2_ (mmHg)	36.7 ± 8.0	35.1 ± 7.3	38.2 ± 8.3	*0.010*
PaO_2_ (mmHg)	103.5 ± 68.6	103.1 ± 69.9	104 ± 67.6	0.934
MV (L/min)	4.4 ± 2.7	4.1 ± 2.4	4.9 ± 3.0	0.079
PIP (cmH2O)	20.8 ± 4.9	21.3 ± 4.5	20.4 ± 5.2	0.223

Values are expressed as mean ± standard deviation; significant *P* values are in italic

*ECMO* extracorporeal membrane oxygenation, *PaCO*_*2*_ partial pressure of carbon dioxide, *PaO*_*2*_ partial pressure of oxygen, *MV* minute ventilation, *PIP* peak inspiratory pressure

Infusion cannulas between 17 and 20 French units (Fr) were mainly used. The mean size of the infusion cannula used in the femoral group was numerically larger compared to the jugular group (Table 4, Additional file 3: Table S2), while the size of the drain cannula was significantly smaller in the femoral compared to jugular group. The mean separation distance between the tips of these two cannulas was 96.0 ± 57.4 mm and showed no difference between the two groups. Initially, the blood flow through the ECMO circuit was similar, but higher blood flow was seen at 24 h after initiation of ECMO in the femoral compared to the jugular group.

**Table 4:** ECMO parameters related to oxygenation

	Total (*n* = 180)	Jugular (*n* = 90)	Femoral (*n* = 90)	*P*
Cannula size (Fr)				
Drain cannula	22.5 ± 2.5	23.6 ± 2.2	21.3 ± 2.3	*<0.001*
Infusion cannula	18.6 ± 2.0	17.6 ± 1.6	19.6 ± 1.9	*<0.001*
Cannula distance (mm)	96.0 ± 57.4	85.6 ± 39.7	102.8 ± 65.9	0.146
ECMO flow (L/min/m^2^)				
At 1 h	2.2 ± 0.5	2.2 ± 0.5	2.2 ± 0.5	0.969
At 4 h	2.0 ± 0.7	1.9 ± 0.6	2.2 ± 0.7	0.061
At 24 h	2.2 ± 0.5	2.1 ± 0.5	2.4 ± 0.5	*0.022*
Blood oxygen content				
PaO_2_ post-oxygenator (mmHg)	459.2 (359.4–535.9)	460.4 (235.5–526.2)	450.5 (411–554.5)	0.250
SaO_2_ post-oxygenator (%)	99.6 (99–99.9)	99.7 (99–99.9)	99.5 (99.2–99.9)	0.650
PaO_2_ pre-oxygenator (mmHg)	46.5 (41–55.1)	49.1 (43.2–57.9)	42.8 (40–49.9)	*0.046*
SaO_2_ pre-oxygenator (%)	81.5 (72.6–87)	82.9 (75.9–89.8)	78.4 (71.1–83.9)	0.110
Difference of blood oxygen content between pre and post-oxygenator (mL/L)	256.9(172.6–390.3)	235.6(150.3–374)	270.3 (234–417.5)	0.061

Values are expressed as mean ± standard deviation or median (interquartile range); significant *P* values are in italic

*ECMO* extracorporeal membrane oxygenation, *PaO*_*2*_ partial pressure of oxygen, *SaO*_*2*_ oxygen saturation

### Clinical outcomes and adverse events related to cannulation

**Fig. 2:**
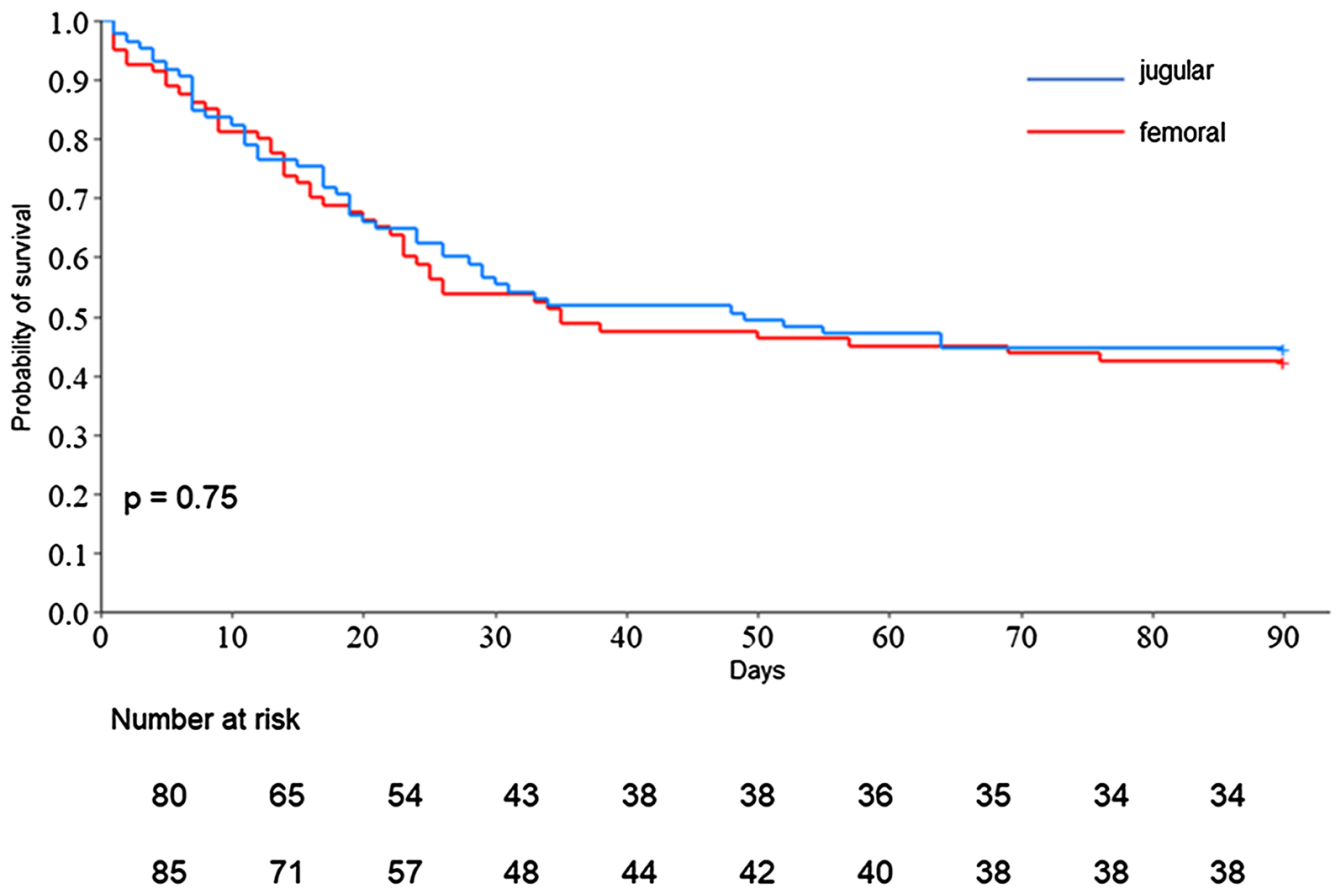
Kaplan–Meier survival curves and 90-day outcome. Red line indicating jugular group in which return cannula site of VV-ECMO is femoral vein. Blue line depicting femoral group in which return cannula site of VV-ECMO is internal jugular vein. The difference between jugular and femoral group was not significant (*p* = 0.75 by the log-rank test)

The 90-day mortality rate was 57.1% in the jugular group and 53.9% in the femoral group (*P *= 0.644; Additional file 3: Table S3). A total of 90 matched pairs had concordant outcomes (Table 5). Figure 2 shows the survival curves of this matched cohort, stratified by the configuration of the catheter. Kaplan–Meier analysis revealed no statistically significant differences between the jugular and femoral groups. There were no significant differences between the groups for other outcome variables; namely, mortality at 180 days, weaning rate, and length of ICU and hospital stay.

**Table 5:** Clinical outcomes according to configuration

	Total (*n* = 180)	Jugular (*n* = 90)	Femoral (*n* = 90)	*P*
Tracheostomy	78 (43.6)	39 (43.3)	39 (43.8)	1.0
ECMO duration (days)	16 ± 18.2	17.6 ± 21.2	14.5 ± 14.6	0.248
Interval MV–ECMO (days)	4.3 ± 7.5	4.4 ± 8.8	4.3 ± 6.1	0.879
Hospital stay (days)	56.1 ± 65.1	57.9 ± 60.9	54.3 ± 69.4	0.714
ICU LOS (days)	24.1 ± 22.2	25.4 ± 25.3	22.8 ± 18.6	0.431
Weaning rate	96 (54.5)	46 (53.5)	50 (55.6)	0.901
In-hospital mortality	112 (62.6)	59 (66.3)	53 (58.9)	0.385
90-day mortality	107 (59.8)	55 (61.8)	52 (57.8)	0.692
180-day mortality	114 (73.5)	59 (76.6)	55 (70.5)	0.496
Cannula-related complications				
ECMO site bleeding	21 (11.7)	16 (17.8)	5 (5.6)	*<0.001*
ECMO cannula manipulation	49 (32)	33 (37.9)	16 (24.2)	*0.015*
Infectious complication	41 (22.8)	25 (27.8)	16 (17.8)	*<0.001*

Values are expressed as mean ± standard deviation, or *n* (%); significant *P* values are in italic

*ECMO* extracorporeal membrane oxygenation, *MV* mechanical ventilation, *ICU LOS* length of stay in intensive care unit

Bleeding at the cannulation site leading to transfusion or intervention was more common in the jugular group than in the femoral group (17.8 vs. 5.6%, respectively; P < 0.001). The rate of bloodstream infection due to ECMO cannulation was lower in the femoral group than in the jugular group (17.8 vs. 27.8%, respectively; P < 0.001).

## Discussion

This multicenter study investigated the difference in oxygenation related to infusion catheter location in VV-ECMO performed for acute respiratory distress syndrome. The arterial oxygen after initiation of ECMO was comparable between the femoral and jugular groups and was consistently observed during GEE analysis of repeated measurements, as well as in the propensity score-matched cohort. Also, there were no significant differences in mortality at 90 days after ECMO initiation between the two groups. However, the incidence of cannula-related complications such as bleeding at cannulation site and the BSI were significantly high in the jugular group compared to the femoral group.

The jugular site is often preferred over the femoral site for infusion catheter insertion during VV-ECMO, based on the risk of recirculation and concerns of reduced oxygenation [12]. However, femoral venous access does not appear to reduce oxygenation compared with jugular access in our study. Consistent with our study, Guervilly et al. [7] reported similar arterial PaO_2_ and arterial oxygen content between fem–jug and fem–fem configuration in a retrospective study with a relatively small sample size.

Despite similar arterial oxygen levels between two groups, the femoral group required higher minute ventilation to maintain comparable oxygenation and showed PaCO_2_ level, compared to the jugular group. However, higher minute ventilation also existed for the femoral group prior to initiation of ECMO, and the minute ventilation pre- and post-ECMO initiation did not change between the two groups. Optimizing gas exchange with ECMO reduces the activation of ventilatory control, allowing lung-protective ventilation and, thus, less ventilator-induced lung injury. In our study, both the driving pressure and peak inspiratory pressure, often used as surrogate marker of lung injury, were not different between the two groups. Furthermore, the robust clinical outcomes related to ventilator-induced lung injuries such as the rate of tracheostomy, ECMO duration, and weaning rate of ECMO did not show any significant differences regardless of the difference in ventilatory support and level of PaCO_2_.

The fem–jug configuration may theoretically enable higher blood flows, as the return cannula directs flow across the tricuspid valve, and has been found to have higher flows than the atrio-femoral configurations [4, 13]. However, a recent retrospective study reported similar blood flows both in the fem–jug and fem–fem configurations [7]. Similarly, the flow rates immediately after and 4 h after ECMO initiation were not different between the femoral and jugular groups in our study. In contrast, the blood flow rate at 24 h post-ECMO initiation was higher in the femoral group compared to the jugular group, suggesting that a relatively higher blood flow rate was necessary for the femoral group to reach adequate arterial oxygenation. However, the ECMO flow rates are usually limited by the size of the cannula which likely explains this significant difference between the two configurations.

Of late, cannulation is performed with a fem–jug configuration due to higher recirculation issues in fem–fem configuration [8, 12]. In the fem–jug configuration, the blood from the infusion cannula flows directly across the tricuspid valve and not toward the drain cannula, possibly mitigating the amount of recirculation. However, proper positioning of the return catheter tip in front of the mitral valve is difficult without transesophageal echocardiography or fluoroscopic guidance. The dual-lumen cannula, inserted with jugular cannulation, also requires the same image guidance for placement, and its malposition could increase the recirculation rates to as high as 50% [14]. Even when positioned properly at the time of insertion, patient factors such as movement from a supine to a seated position or rotation of the head and neck could affect the orientation of the cannula, thereby affecting the amount of recirculation [15].

Recent studies have used a return cannula inserted via femoral vein with a multistage draining cannula inserted in the jugular vein to minimize recirculation [12, 16]. Although authors investigated patients with draining cannula introduced through the jugular vein, they found reinfusing oxygenated blood via femoral vein with effective drainage cannula would not precipitate recirculation, mitigating oxygen delivery during VV-ECMO. In addition, increasing the distance between the drainage and infusion cannulas is one of the most direct ways to reduce the amount of recirculation in VV-ECMO [5]. Burrell et al. [5] concluded that recirculation is rarely a problem if the cannulas tips are separated ≥ 8 cm in the inferior vena cava (IVC). Accordingly, the tip-to-tip distance between the two cannulas was more than 8 cm in both femoral and jugular group in our study.

Unexpectedly, the jugular group showed a higher incidence of cannula-related complications, including ECMO site bleeding and catheter-related BSI compared to the femoral group. The advantage of femoral vein cannulation is that the site is almost always accessible and requires less skill for insertion than jugular vein cannulation [4]. Adverse effects such as bleeding after femoral vein cannulation can usually be controlled with local pressure. The largest multicenter trial published to date reported lower mechanical complications with femoral vein cannulation compared to internal jugular vein access, with complications defined as bleeding requiring transfusion of at least two units of blood, or hematoma requiring transfusion or operative intervention [17].

A recent multicenter study found no difference in catheter-related bloodstream infection or major catheter-related infection between the internal jugular and femoral vein for central venous catheterization [18]. A meta-analysis also failed to demonstrate any significant difference in infectious risk between the femoral and internal jugular sites [19]. In a multicenter randomized trial, jugular vein catheterization access did not reduce the risk of infection compared to femoral access, except among adults with a high body mass index (BMI) [20]. The relatively low BMI of our study population could explain the lower incidence of catheter-related bloodstream infections in the femoral group.

Furthermore, the frequency of manipulation was higher, with jugular cannula in this study. Unlike the femoral veins, proper positioning of jugular catheters requires transesophageal echocardiography or fluoroscopic guidance, which adds to the complexity of cannulation and may result in more attempts to manipulate catheter position, during ECMO support. Manipulation of catheters can expose the patient to non-sterile parts of the cannula and increase the risk of infection [4]. A higher rate of manipulation in jugular group could contribute to the increased incidence of catheter-related BSI in our study.

The in-hospital mortality rate of 62% in our study is higher than the reported rate of 42% in the ELSO registry [21]. The relatively higher mortality rate may be due to excessive use of ECMO in patients who may have shown good response to NMB and prone positioning. The proportion of prone position before ECMO therapy was very low compared to that in the EOLIA trial [8], in which prone positioning was applied in 90% of patients in the conventional ventilator support group, with a survival rate of 54%. In addition, a higher proportion of the elderly population in our study may explain the difference in the mortality rates. The mean age of patients who received ECMO in our study was 55 years, which is higher than that of patients included in the ELSO registry. Consistent with our findings, an ECMO epidemiologic study performed in Germany showed that the mortality rate of patients was approximately 60%, and that 80% of patients who received ECMO therapy were older than 40 years [22].

The current study has some limitations. First, this was a retrospective observational study, and although we used the propensity score matching to control for selection bias, the effects of confounding factors may not have been entirely excluded. In particular, factors such as overall fluid balance including input and output or any inclusion or exclusion criteria would also be important confounding variables, but due to the limitation of multicenter retrospective studies, it is difficult to collect additional information. Another limitation is that our study population had a relatively lower body mass index compared to the Western population, limiting the generalization of our findings to different races. However, in Koreans, VV-ECMO with fem–fem configuration does not appear inferior to fem–jug, and more ideally in the Asian population if dual-lumen cannulas are not available or are deemed unsuitable. Although dual-lumen cannulas are gaining popularity, dual-lumen jugular cannulation has been reported to have a higher rate of venous thrombosis than single-lumen femoral cannulation [23] and the increment in its utilization has not been shown in the ELSO Registry International Report [ELSO International Report, January 2017, unpublished data] [24]. Third, we did not quantify recirculation occurring during VV-ECMO with an ultrasound flow detection device. Cardiac output was also not determined, which leaves the possibility that a sufficient ratio of ECMO flow to cardiac output for oxygenation was not achieved in some patients with fem–jug configuration. However, we measured the oxygen content indicating that acceptable arterial oxygenation could be delivered with either configuration.

## Conclusions

In conclusion, the fem–jug and fem–fem configurations showed comparable clinical outcomes in terms of short-term oxygenation in patients with ARDS managed with VV-ECMO. However, the incidence of ECMO-related complications was higher with the fem–jug configuration. Future well-designed randomized control trials are required to confirm and supplement our findings.

## Supplementary information

**Additional file 1.** Patient flow diagram. ECMO: extracorporeal membrane oxygenation, VA-ECMO: Venous-arterial ECMO, APACHE: Acute Physiology and Chronic Health Evaluation, SOFA: Sequential Organ Failure Assessment, RESP: Respiratory ECMO Survival Prediction, CRRT: continuous renal replacement therapy.

**Additional file 2.** Standardized differences in the mean or proportion of variables before and after matching. SOFA: Sequential Organ Failure Assessment, RESP: Respiratory ECMO Survival Prediction, NO: nitric oxide, NMB: neuromuscular blockade, MV: mechanical ventilation, Ex: Etiology of respiratory failure, CRRT: continuous renal replacement therapy, APACHE: Acute Physiology and Chronic Health Evaluation.

**Additional file 3: Table S1.** Oxygenation and Ventilation profiles during ECMO (unmatched cohort). **Table S2.** ECMO parameters related to oxygenation (unmatched cohort)**. Table S3.** Clinical outcomes according to configuration (unmatched cohort).

## Data Availability

The datasets used and analyzed during the current study are available from the corresponding author on reasonable request.

## Abbreviations

VV-ECMO: Veno-venous extracorporeal membrane oxygenation
ARDS: Acute respiratory distress syndrome
PaO2: Partial pressure of oxygen
SVC: Superior vena cava
APACHE: Acute Physiology, and Chronic Health Evaluation
SOFA: Sequential Organ Failure Assessment
ICU: Intensive care unit
CRRT: Continuous renal replacement therapy
BSI: Bloodstream infection
ELSO: Extracorporeal Life Support Organization
GEE: Generalized estimating equations
RESP: Respiratory ECMO Survival Prediction
FiO2: The fraction of inspired oxygen
PaCO2: Partial pressure of carbon dioxide
IVC: The inferior vena cava
BMI: Body mass index
