# Supplementary figures and images for: Clinical outcomes according to cannula configurations in patients with acute respiratory distress syndrome under veno-venous extracorporeal membrane oxygenation: a Korean multicenter study
Source: Ann Intensive Care. 2020 Jun 22;10:86. doi: 10.1186/s13613-020-00700-9 (PMC7306930; doi:10.1186/s13613-020-00700-9)

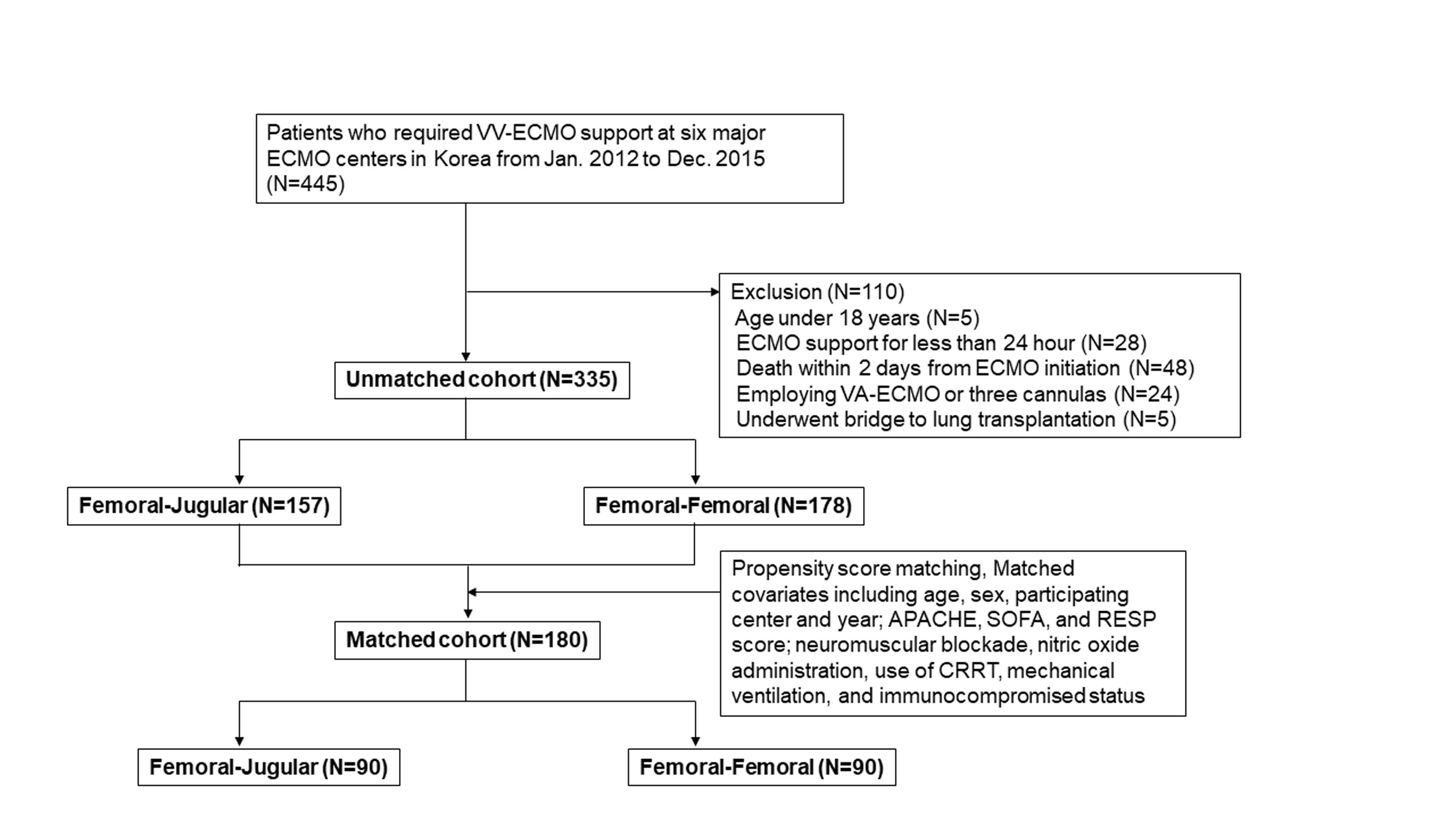

Supplement: Supplementary file 1 — Additional file 1. Patient flow diagram. ECMO: extracorporeal membrane oxygenation, VA-ECMO: Venous-arterial ECMO, APACHE: Acute Physiology and Chronic Health Evaluation, SOFA: Sequential Organ Failure Assessment, RESP: Respiratory ECMO Survival Prediction, CRRT: continuous renal replacement therapy. [file 13613_2020_700_MOESM1_ESM.tif]

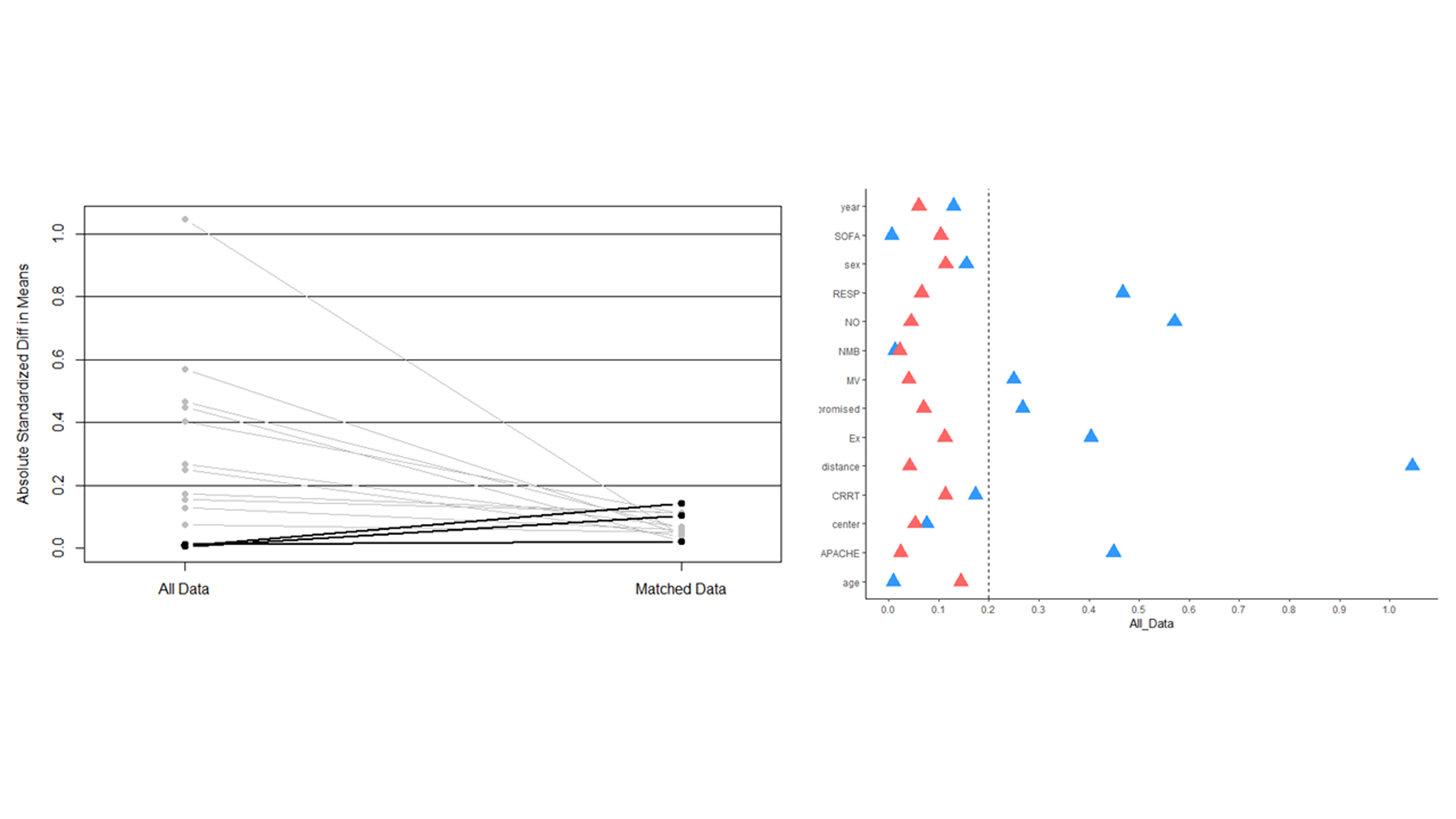

Supplement: Supplementary file 2 — Additional file 2. Standardized differences in the mean or proportion of variables before and after matching. SOFA: Sequential Organ Failure Assessment, RESP: Respiratory ECMO Survival Prediction, NO: nitric oxide, NMB: neuromuscular blockade, MV: mechanical ventilation, Ex: Etiology of respiratory failure, CRRT: continuous renal replacement therapy, APACHE: Acute Physiology and Chronic Health Evaluation. [file 13613_2020_700_MOESM2_ESM.tif]
