# Supplementary material for: Clinical outcomes according to cannula configurations in patients with acute respiratory distress syndrome under veno-venous extracorporeal membrane oxygenation: a Korean multicenter study
Source: Ann Intensive Care. 2020 Jun 22;10:86. doi: 10.1186/s13613-020-00700-9 (PMC7306930; doi:10.1186/s13613-020-00700-9)
Supplement: Supplementary file 3 — Additional file 3: Table S1. Oxygenation and Ventilation profiles during ECMO (unmatched cohort). Table S2. ECMO parameters related to oxygenation (unmatched cohort). Table S3. Clinical outcomes according to configuration (unmatched cohort). [file 13613_2020_700_MOESM3_ESM.docx]

**Table 1 Oxygenation and Ventilation profiles during ECMO (unmatched cohort)**

|  |  | Total  (*n* = 335) | Jugular  (*n* = 157) | Femoral  (*n* = 178) | *P* |
| --- | --- | --- | --- | --- | --- |
| Baseline | PaCO_2_ (mmHg) | 55.6 ± 23.7 | 54.9 ± 25.4 | 56.3 ± 22.0 | 0.620 |
|  | PaO_2_ (mmHg) | 67.8 ± 31.9 | 67.0 ± 24.5 | 68.7 ± 37.6 | 0.641 |
|  | MV (L/min) | 10.3 ± 4.4 | 9.0 ± 3.9 | 11.7 ± 4.5 | **<0.001** |
|  | PIP (cmH2O) | 28.8 ± 6.5 | 28.1 ± 6.7 | 29.6 ± 6.1 | **0.006** |
| Immediately after cannulation | PaCO_2_ (mmHg) | 35.0 ± 11.5 | 33.1 ± 11.5 | 36.7 ± 11.3 | **0.004** |
|  | PaO_2_ (mmHg) | 169.4 ± 146.7 | 153.6 ± 121.6 | 183.1 ± 164.6 | 0.066 |
|  | MV (L/min) | 5.5 ± 3.8 | 4.3 ± 2.8 | 6.7 ± 4.3 | **<0.001** |
|  | PIP (cmH2O) | 22.6 ± 5.9 | 21.8 ± 5.3 | 23.3 ± 6.3 | 0.077 |
| 4 hours after cannulation | PaCO_2_ (mmHg) | 34.1 ± 8.2 | 32.2 ± 8.2 | 35.8 ± 7.9 | **0.000** |
|  | PaO_2_ (mmHg) | 103.8 ± 60.9 | 102.8 ± 64.0 | 104.7 ± 58.1 | 0.788 |
|  | MV (L/min) | 4.3 ± 2.7 | 3.6 ± 2.3 | 5.0 ± 2.9 | **<0.001** |
|  | PIP (cmH2O) | 21.6 ± 4.8 | 21.6 ± 4.4 | 21.7 ± 5.2 | 0.821 |
| 24 hours after cannulation | PaCO_2_ (mmHg) | 36.7 ± 7.5 | 35.3 ± 7.1 | 37.9 ± 7.7 | **0.002** |
|  | PaO_2_ (mmHg) | 101.6 ± 61.4 | 98.3 ± 56.1 | 104.5 ± 65.7 | 0.362 |
|  | MV (L/min) | 4.3 ± 2.7 | 3.7 ± 2.3 | 4.9 ± 2.8 | **<0.001** |
|  | PIP (cmH2O) | 21.3 ± 4.9 | 21.4 ± 4.5 | 21.2 ± 5.3 | 0.741 |

Values are expressed as mean ± standard deviation; significant *P* values are in bold

ECMO: extracorporeal membrane oxygenation, PaCO_2_: partial pressure of carbon dioxide, PaO_2_: partial pressure of oxygen, MV: minute ventilation, PIP: peak inspiratory pressure

**Table 2 ECMO parameters related to oxygenation (unmatched cohort)**

|  | Total  (*n* = 335) | Jugular  (*n* = 157) | Femoral  (*n* = 178) | *P* |
| --- | --- | --- | --- | --- |
| **Cannula size (Fr)** |  |  |  |  |
| Drain cannula | 22.4 ± 2.6 | 23.6 ± 2.1 | 21.1 ± 2.5 | **<0.001** |
| Infusion cannula | 18.7 ± 2.0 | 17.7 ± 1.7 | 19.6 ± 1.7 | **<0.001** |
| Cannula distance (mm) | 103.3 ± 62.5 | 88.4 ± 43.2 | 111.7 ± 69.9 | **0.003** |
| **ECMO flow (L/min/m^2^)** |  |  |  |  |
| At 1 hour | 2.2 ± 0.5 | 2.2 ± 0.5 | 2.2 ± 0.5 | 0.914 |
| At 4 hours | 2.0 ± 0.6 | 1.8 ± 0.5 | 2.2 ± 0.6 | **0.007** |
| At 24 hours | 2.1 ± 0.5 | 2.1 ± 0.5 | 2.2 ± 0.6 | 0.062 |
| **Blood Oxygen content** |  |  |  |  |
| PaO_2_ post-oxygenator (mmHg) | 390.4 ± 154.3 | 369.3 ± 171.6 | 431.2 ± 103.1 | 0.142 |
| SaO_2_ post-oxygenator (%) | 99.4 ± 0.9 | 99.3 ± 1.0 | 99.4 ± 0.7 | 0.322 |
| PaO_2_ pre-oxygenator (mmHg) | 49.2 ± 13.1 | 51.7 ± 14.0 | 45.1 ± 10.2 | **0.001** |
| SaO_2_ pre-oxygenator (%) | 78.8 ± 9.5 | 80.7 ± 9.6 | 75.7 ± 8.4 | **0.002** |
| Difference of blood oxygen content between pre and post-oxygenator (mL/L) | 270.9(165-368.9) | 334.3.6(243.5-434) | 284.8(195-387.5) | **0.026** |

Values are expressed as mean ± standard deviation or median (interquartile range); significant *P* values are in bold

ECMO: extracorporeal membrane oxygenation, PaO_2_: partial pressure of oxygen, SaO_2_: oxygen saturation

**Table 3 Clinical outcomes according to configuration (unmatched cohort)**

|  | Total  (*n* = 335) | Jugular  (*n* = 157) | Femoral  (*n* = 178) | *P* |
| --- | --- | --- | --- | --- |
| Tracheostomy | 144 (45.0%) | 69 (47.9%) | 75 (42.6%) | 0.403 |
| ECMO duration (days) | 15.5 ± 17.0 | 17.5 ± 18.9 | 13.7 ± 14.9 | **0.041** |
| Interval MV–ECMO (days) | 4.8 ± 7.8 | 4.6 ± 8.7 | 4.9 ± 6.9 | 0.751 |
| Hospital stay (days) | 54.6 ± 62.0 | 53.3 ± 51.7 | 55.8 ± 70.0 | 0.712 |
| ICU LOS (days) | 24.1 ± 22.3 | 26.3 ± 24.4 | 22.1 ± 20.2 | 0.092 |
| Weaning rate | 180 (55.7%) | 82 (56.6%) | 98 (55.1%) | 0.876 |
| In-hospital mortality | 199 (59.9%) | 92 (59.7%) | 107 (60.1%) | 1.000 |
| 90-day mortality | 185 (55.4%) | 89 (57.1%) | 96 (53.9%) | 0.644 |
| 180-day mortality | 203 (81.5%) | 96 (82.1%) | 107 (81.1%) | 0.970 |
| Cannula related complications |  |  |  |  |
| ECMO site bleeding | 38 (11.4%) | 22 (14.1%) | 16 (9.0%) | 0.195 |
| ECMO cannula manipulation | 84 (28.6%) | 51 (33.8%) | 33 (23.1%) | 0.057 |
| Infectious complication | 71 (21.3%) | 46 (29.5%) | 25 (14.0%) | **0.001** |

Values are expressed as mean ± standard deviation, or *n* (%); significant *P* values are in bold

ECMO: extracorporeal membrane oxygenation, MV: mechanical ventilation, ICU LOS: length of stay in intensive care unit
